# Finite element analysis of stress in oral mucosa and titanium mesh interface

**DOI:** 10.1186/s12903-022-02703-3

**Published:** 2023-01-17

**Authors:** Chen-Xi Wang, Qi-Guo Rong, Ning Zhu, Ting Ma, Yu Zhang, Ye Lin

**Affiliations:** 1 Department of Oral Implantology, Peking University School and Hospital of Stomatology and National Clinical Research Center for Oral Diseases and National Engineering Laboratory for Digital and Material Technology of Stomatology and Beijing Key Laboratory of Digital Stomatology, Beijing, 100081 China; 2 College of Engineering, Peking University, Beijing, 100871 China

**Keywords:** KeGuide bone regeneration, Titanium mesh exposure, Oral mucosa, Finite element analysis

## Abstract

**Background:**

The stiffness of titanium mesh is a double-blade sword to repair larger alveolar ridges defect with excellent space maintenance ability, while invade the surrounding soft tissue and lead to higher mesh exposure rates. Understanding the mechanical of oral mucosa/titanium mesh/bone interface is clinically meaningful. In this study, the above relationship was analyzed by finite elements and verified by setting different keratinized tissue width in oral mucosa.

**Methods:**

Two three-dimensional finite element models were constructed with 5 mm keratinized tissue in labial mucosa (KM cases) and 0 mm keratinized tissue in labial mucosa (LM cases). Each model was composed of titanium mesh, titanium screws, graft materials, bone, teeth and oral mucosa. After that, a vertical (30 N) loadings were applied from both alveolar ridges direction and labial mucosa direction to stimulate the force from masticatory system. The displacements and von Mises stress of each element at the interfaces were analyzed.

**Results:**

Little displacements were found for titanium mesh, titanium screws, graft materials, bone and teeth in both LM and KM cases under different loading conditions. The maximum von Mises stress was found around the lingual titanium screw insertion place for those elements in all cases. The keratinized tissue decreased the displacement of oral mucosa, decreased the maximum von Mises stress generated by an alveolar ridges direction load, while increased those stress from labial mucosa direction load. Only the von Mises stress of the KM cases was all lower than the tensile strength of the oral mucosa.

**Conclusion:**

The mucosa was vulnerable under the increasing stress generated by the force from masticatory system. The adequate buccal keratinized mucosa width are critical factors in reducing the stress beyond the titanium mesh, which might reduce the titanium exposure rate.

## Background

Prevalence of tooth loss has increased due to globe population aging. Tooth loss negatively affects the overall physical and social well-being of older adults [1]. Besides, tooth loss could also induce the secondary absorption and atrophy of alveolar bone. Therefore, the reconstruction of alveolar bone always plays an important role for teeth restoration. Previously research has reported several clinical technique to repair alveolar bone defects, guided bone regeneration technique (GBR) is currently one of the most used method due to its simple operation and osteogenic stability [2]. The effect of GBR is related to the performance of the barrier membrane used to separate soft and hard tissues [3]. The barrier membrane could be divided into absorbable and nonabsorbable membrane. Collagen membrane is the most common used absorbable barrier membrane. However, it has been reported the collagen membrane was soft to maintain the space for large bone defects. With the development of additive manufacturing, titanium mesh shows superior mechanical properties and great osteogenic performance during application [4].

Previously research has demonstrated the wound dehiscence and titanium mesh exposure are the main complications in GBR with titanium mesh [5]. The incidence of mesh exposure is mostly between 20 and 30% and the highest reported exposure rate is 66% [6, 7]. Currently, several studies has shown the application of custom-made titanium mesh through the digital process decreasing the exposure rate by avoiding most sharp edges caused by intraoperative bending [8]. Other studies believed the use of platelet-rich fibrin (PRF) was an effective method [9]. However, those reports often contained a small sample size. There is a meta-analysis has compared the exposure rate of customized and conventional titanium mesh in GBR surgery, and the results showed the customized titanium mesh had a lower rate (31%) than conventional titanium mesh (51%) [10]. However, another meta-analysis showed the exposure rate was similar between customized and conventional titanium mesh [11]. The same article demonstrated the type of bone graft material and the use of absorbable membranes in surgical procedure had little effect in preventing titanium mesh exposure, which is in consistency with another in-vivo experiments [12]. Besides, different advancement surgical techniques also showed no correlation with mesh exposure area and rate [13].

Therefore, the reason for titanium mesh exposure needs further research. Infection is one of the certain factors, but not all of the surgical areas infection after exposure [14]. Besides, the areas are continued stimulated by mechanical force from food intake or oral hygiene maintenance, therefore, we hypothesis the stress distribution on oral mucosa play an important role in preventing titanium mesh exposure. Currently, the finite element method is an efficiency and reliable method for studying the stress distribution between interfaces [15]. Compared with the traditional photoelasticity method, it has the advantages of accuracy and stability. However, the titanium mesh always fabricated into porous structures to enhance vascularization in areas of bone regeneration. And, as an elastomer, the oral mucosa tends to generate deformation to squeeze the underlying materials and tissues. To our best knowledge, there are still few studies on the interface stress between the elastic oral mucosa and porous titanium mesh. Therefore, this study is aimed to build a finite element model to analyze the stress distribution between titanium mesh and oral mucosa under different loading condition. And verify the simulation of the model by analyzing different situation of oral mucosa.

## Materials and methods

### Model design

**Fig. 1:**
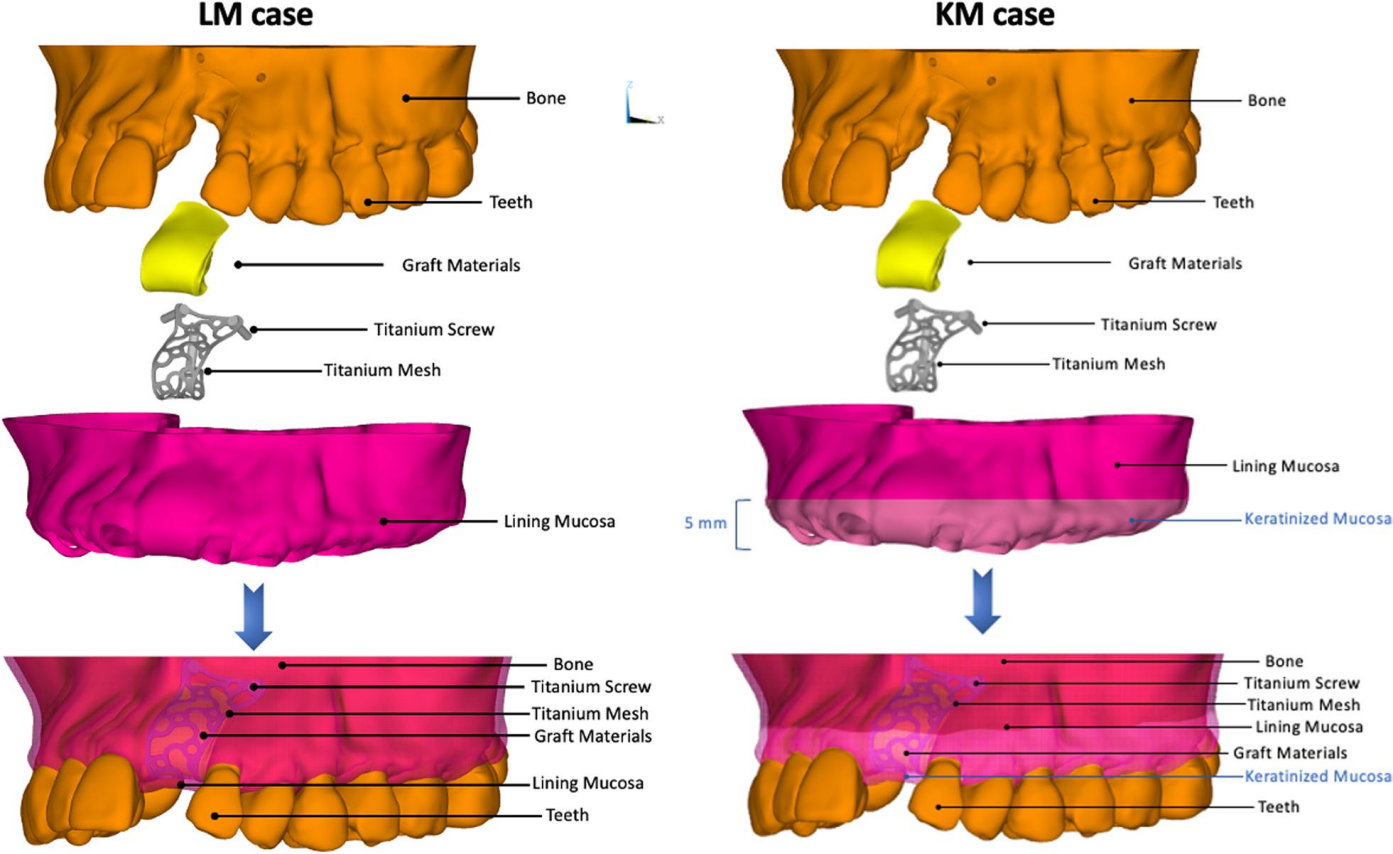
Finite element models used in the present study. Both two models contain the elements of bone, teeth, graft materials, titanium mesh and screws and lining mucosa. Compared to the LM case, the KM case has additional 5 mm keratinized mucosa in buccal (the blue lines indicates the mainly different between two cases)

The present study was approved by the Clinical Research Ethics Committee of the Peking University School and Hospital of Stomatology (Grant No. PKUSSIRB-202056085). The 3D geometries of the maxillary came from a cone beam computerized tomography (CBCT) file of a maxillary bone defect patient. It was confirmed that the subject informed consent to the collection and the date was used in our 3D model reconstruction. The CT images were (slice thickness 0.5 mm, pixel size 0.398 mm) served as the DICOM file for reconstruct a 3D model in Mimics software (Materialise, Leuven, Belgium). Since the spongy bone in the anterior region is denser and this study mainly focus on the interface between oral mucosa and titanium mesh, to simplify the modeling process, we didn’t differentiate between cortical and spongy bone. The bone graft material that filled in the above bone defects was generated using 3Matic software (Materialise, Leuven, Belgium). The titanium mesh and screws were design based on the morphology of virtual bone augmentation by SolidWorks 16.0 (SolidWorks Corporation, VelizyVillacoublay, France). The thickness of the titanium mesh was set as 0.3 mm and the titanium screws were designed as cylinders with a length of 5 mm in the buccal side of the model and 7 mm in palatal. The mucosa covering the maxillary was also generated and assembled with bone, titanium mesh, bone graft material using Geomagic Studio software (Geomagic Company, NC, USA). After that, the 3D model was meshed and calculated (Fig. 1a).

Two three-dimensional finite element models were constructed using the FE software ANSYS 16.0 (Swanson Analysis System Co., Houston, TX, USA) by considering different keratinized mucosa covering types (LM case: buccal-0.0 mm and lingual-5.0 mm, KM case: buccal-5.0 mm and lingual-5.0 mm) (Fig. 1). After that, the 10-noded tetrahedral element was used to mesh the model. In addition, fine meshing was performed in the region of interest.

Table 1 summarizes the material properties of the model components, including bone, bone graft materials, teeth, lining mucosa, keratinized mucosa and titanium alloy, which were taken from the literature [16]. All of the materials were considered linearly elastic, homogenous, and isotropic. Table 2 lists the total number of elements and nodes for each model.

**Table 1:** Mechanical properties of the finite element model components

Material	Young’s modulus (MPa)	Poisson’s ratio
Bone [17, 18]	13,700	0.3
Graft materials [19, 20]	1000	0.3
Teeth [19, 21]	20,000	0.3
Titanium alloy [16, 18, 19]	110,000	0.3
Lining mucosa [22, 23]	10	0.3
Keratinized mucosa [22, 23]	50	0.3

**Table 2:** Numbers of tetrahedral elements of each finite element model component

Mucosa type	Bone	Teeth	Graft materials	Mesh	Screws	Keratinized mucosa	Lining mucosa
LM	328,273	303,298	43,328	97,423	22,943	0	3,241,331
KM	327,132	301,224	41,432	96,254	21,922	1,103,492	2,014,582

### Contact management and loading conditions

**Fig. 2:**
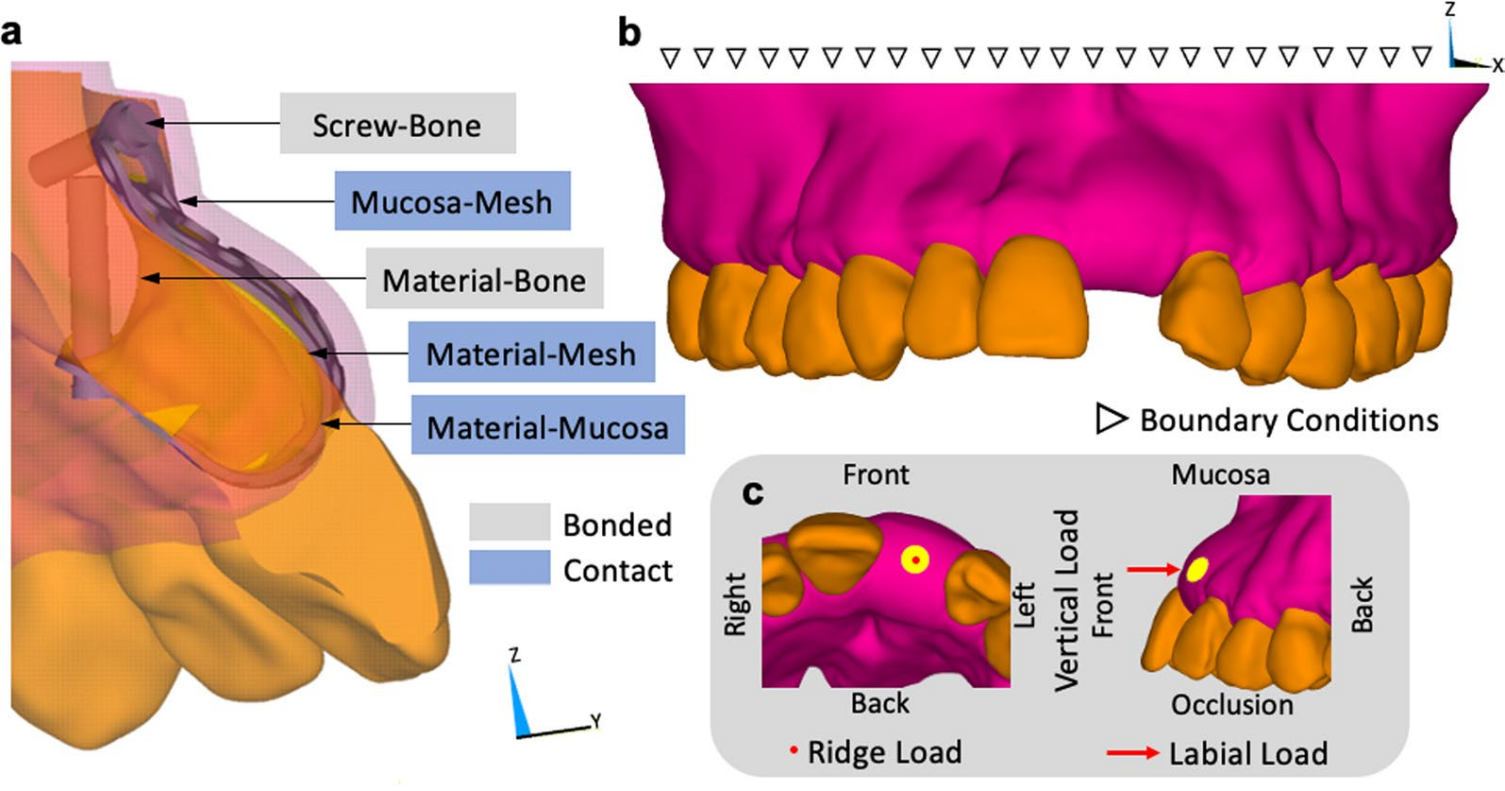
Finite element model parameters. **a** contact surfaces for all the elements. **b** boundary and **c** loading conditions

The titanium mesh, screws and bone interface were defined as a “bonded” to stimulate the effect of fixation titanium screws. The interfaces between oral mucosa, titanium mesh and grafting materials were defined as “contact” to analyze the displacements of oral mucosa. The friction coefficients were both 0.2 for the oral mucosa/titanium mesh, and titanium mesh/ graft materials interfaces (Fig. 2a). Set the maxillary marginal bone as the boundary to limit the movement of the models, where is considered to be firmly attached to the skull (Fig. 2b). A 30 N loading force on the highest 100 nodes was vertically applied on both the occlusal and labial side of the mucosa on titanium mesh to simulate non-physiological loads from food intake and maintenance of oral hygiene (Fig. 2c) [24]. With the light force applied on the model, a small deformation was considered in the study. The data regarding stress and displacement of the mucosa were outputted for further analysis.

## Results

**Fig. 3:**
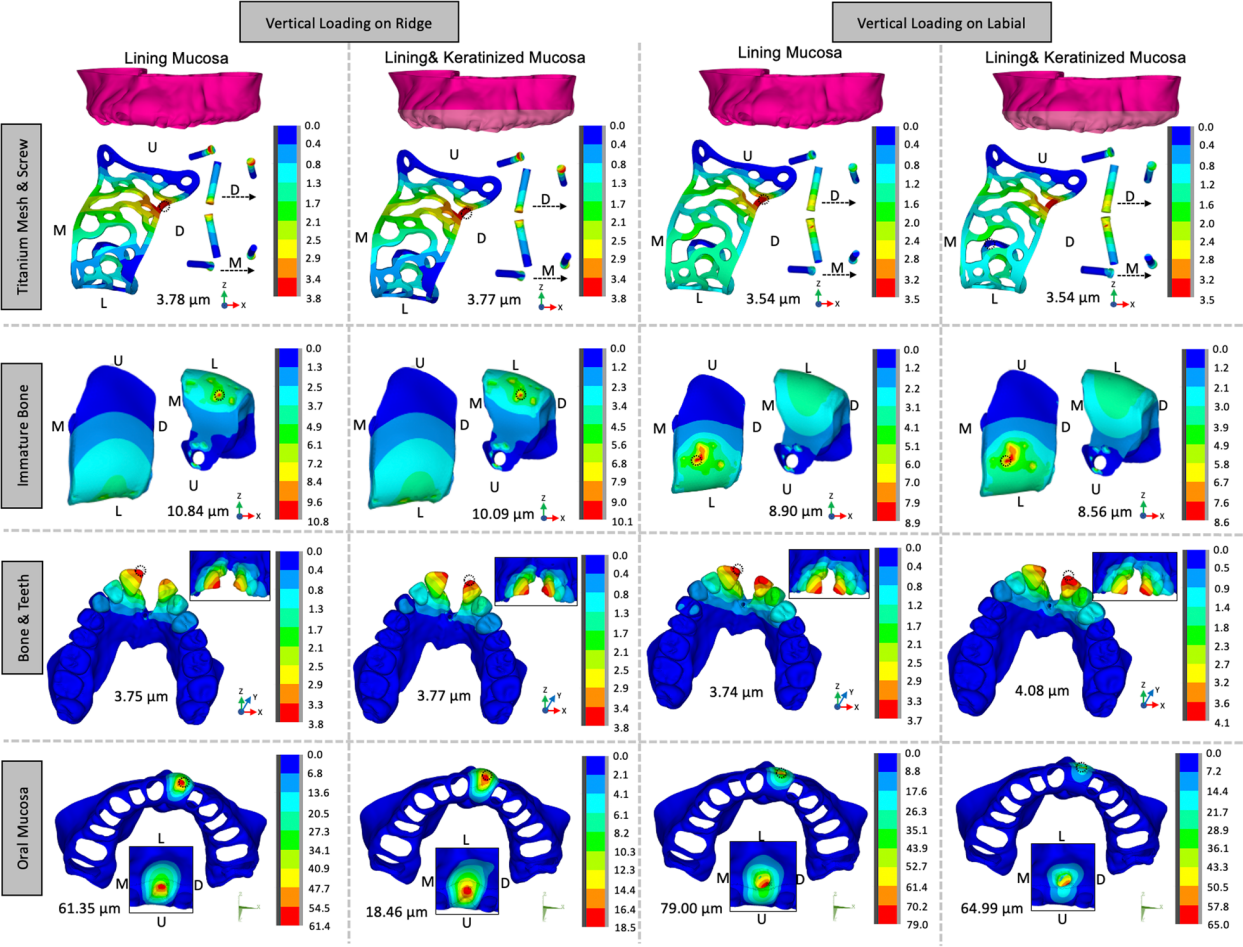
The displacement pattern of all the components under occlusal or labial loading. Light pink regions indicate the keratinized mucosa on titanium mesh (*G* gingival direction, *O* occlusal direction, *M* mesial direction, *D* distal direction): **a** the displacement of titanium mesh and screws, **b** the displacement of graft materials, **c** the displacement of bone and teeth, and **d** the displacement of oral mucosa

Figure 3 shows the displacement patterns of titanium mesh/screws, graft materials, bone/teeth and oral mucosa in LM and KM cases under vertical loading from both occlusal and labial sides. The displacement patterns were tiny and similar in all models except the oral mucosa, which indicated the perfect space maintenance ability of titanium mesh regardless of the characteristics of the mucosa and the direction of the load [25]. For the oral mucosa, the displacement formation area was observed around the loading area for all the models. The displacement was the smallest for the KM case with vertical loading on ridge (18.46 μm) and the largest for LM case with vertical loading on labial (79.00 μm). The keratinized mucosa significantly decreased the initial displacement of oral mucosa.

**Fig. 4:**
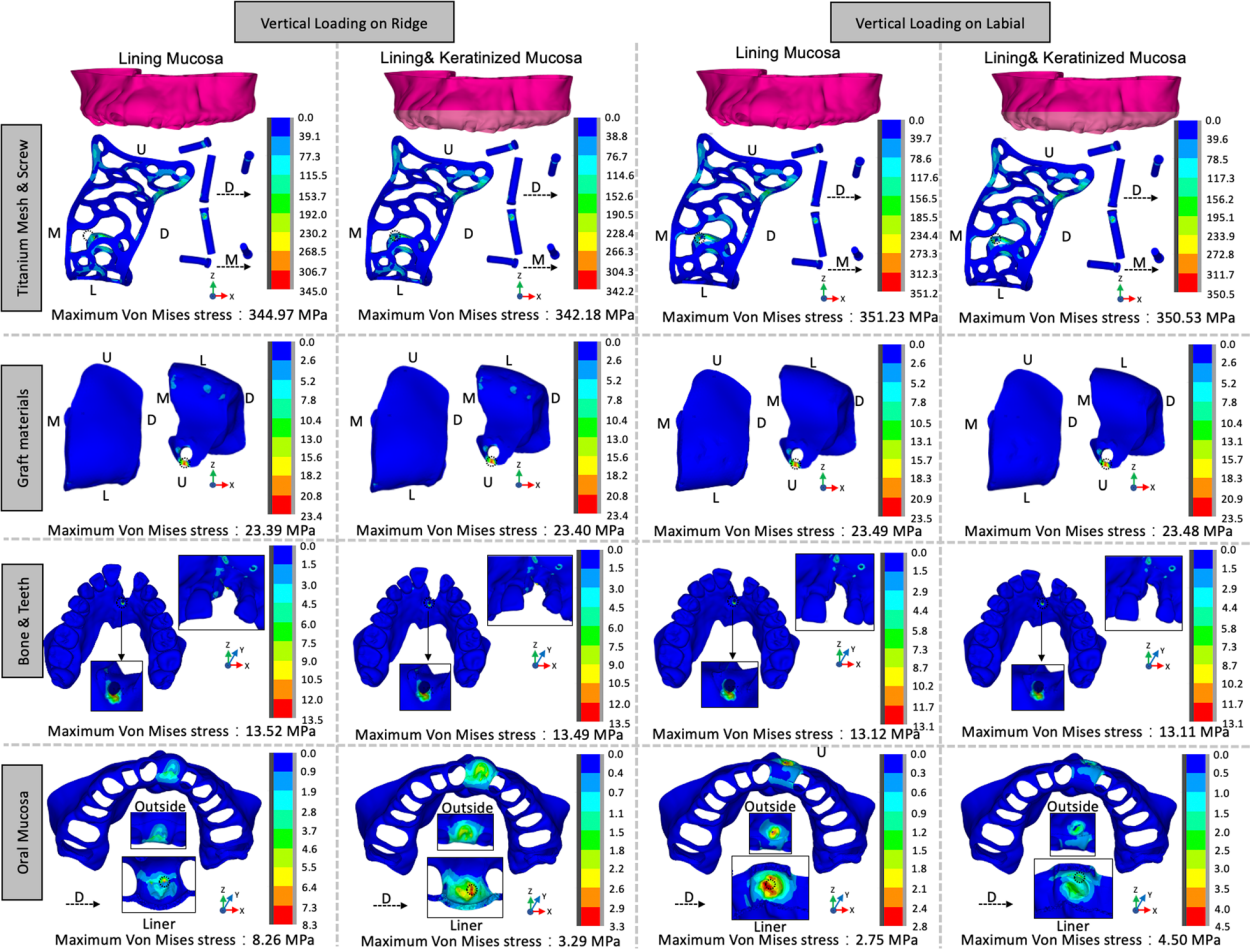
The von Mises stress distribution of all the components under occlusal or labial loading. Light pink regions indicate the keratinized mucosa on titanium mesh. (*G* gingival direction, *O* occlusal direction, *M* mesial direction, *D* distal direction): **a** the von Mises stress distribution of titanium mesh and screws, **b** the von Mises stress distribution of graft materials, **c** the von Mises stress distribution of bone and teeth, and **d** the von Mises stress distribution of oral mucosa

Figure 4 displays the von Mises stress distribution for all the components in each model. The stress distribution patterns were similar in titanium mesh, titanium screws, graft materials, bone and teeth for all models, where the maximum von Mises stress was mainly focus on the lingual titanium screw insertion place. For the oral mucosa, the maximum von Mises stress was detected in the liner side in all models. The keratinized mucosa increased maximum von Mises stress of oral mucosa from 2.75 to 4.50 MPa following a ridge direction 30 N load and decreased maximum von Mises stress from 8.26 to 3.29 MPa following a labial direction 30 N load. In general, the keratinized mucosa expanded the range of von Mises stress distribution and reduces the tendency of stress concentration, which was thought to be benefit in reducing soft tissue complications of titanium mesh.

**Fig. 5:**
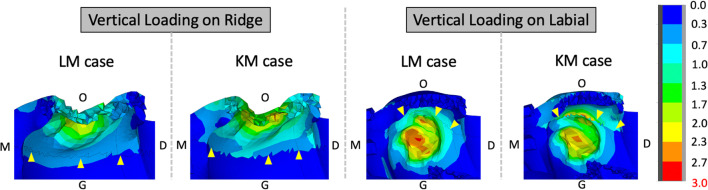
The von Mises stress distribution of keratinized mucosa and lining mucosa under occlusal or labial loading (*G* gingival direction, *O* occlusal direction, *M* mesial direction, *D* distal direction): the yellow arrows indicate the dividing line of keratinized mucosa and lining mucosa

To further compared the von Mises stress in each model, we unify the maximum stress in the gauge bar to 3 MPa base on a previously research reported the tensile strength of oral mucosa was 3.81 ± 0.9 MPa [23]. Considered most titanium mesh exposure at the labial/buccal side of oral mucosa [26], Fig. 5 presents the von Mises stress on the labial section of mucosa contact with titanium mesh. The existent of keratinized mucosa made the von Mises stress distribution more uniform on mucosa after loading on different directions. The loading from labial direction generated more yellow and red areas on lining mucosa, indicating a higher von Mises stress jeopardizing the health of soft tissue. The keratinized mucosa induced the maximum von Mises stress shifting to the occlusal direction and reduced the maximum von Mises stress to 2.50 MPa on lining mucosa compared to no keratinized mucosa case with a 2.75 MPa maximum von Mises stress.

## Discussion

The exposure of titanium mesh is the most common complication. Based on the exposure time, the exposure of titanium mesh can be divided into early exposure and late exposure [8]. Previously research has shown that the late exposure of titanium mesh that occurred 4 weeks after bone augmentation may cause 15–25% of the graft resorption in the exposed area and decrease the volume of new bone formation [27]. Therefore, in this study, we have set all the graft materials below the titanium mesh in an immature bone physical property (1000 MPa) to stimulate the new bone formation in late exposure of titanium mesh. Besides, based on the bone regeneration features, the contact conditions of graft materials and original bone was set to “bonded”. Since the titanium mesh was inserted into oral system, a continuous stress, especially after initial soft tissue healed, was generated on the soft and hard tissues around the titanium mesh under the constant force from masticatory system. The titanium mesh was submerged healing under the oral mucosa and didn’t contact with the opposite teeth directly, therefore, a 30 N vertical loading force was applied from both occlusal and labial sides of the oral mucosa to simulate masticate, which is in consistency with previously research [28].

The finite element models in this study were established based on an actual participant carrying on bone augmentation with titanium mesh. The clinical outcomes showed great space maintenance ability of titanium mesh with no complication like mesh rupture. In consistency with the clinical observation, based on the above parameter settings, our stimulation results showed the maximum von Mises stress value of the titanium mesh was 351.23 MPa which was only 20–30% of the yield strength of titanium alloy (σ = 780–950 MPa) [29]. Besides, the displacement of graft materials was rare, the maximal value was 10.84 μm occurred at alveolar crest area in LM case under the occlusal direction load, which indicated the perfect space maintaining ability of titanium mesh. A micron level displacement was comparable to another maxillary anterior titanium mesh insertion model [30]. The above results manifested the simulation performance of the model. However, the maximum von Mises stress value of the titanium mesh in all models was 8.29 MPa which was nearly 2 times higher than tensile strength of oral mucosa (σ = 1–4.5 MPa) and indicated the constant force from masticatory system might be a risk of titanium mesh exposure [23, 31].

Previously research has shown the keratinized mucosa could resist the mechanical irritation in the oral cavity [32]. Herford et al. used titanium mesh for bone augmentation n in pigs, and the results shown that less titanium mesh exposure rate was found in the group with higher keratinized mucosa width [33]. However, if the less titanium mesh exposure rate was related to better mechanical behavior of keratinized mucosa was hard to investigate in clinical due to several ethical issues. Based on the finite element we built in this study to analyze the stress distribution between oral mucosa and titanium mesh., we have also measured the influence of different keratinized mucosa width on the stress distribution. The result might provide a more intuitive understanding of the relationship between stress distribution and titanium exposure. Ono et al. have proposed a classification of keratinized mucosa around dental implants [34]. The adequate keratinized mucosa was defined as more than 5 mm of keratinized mucosa from the implant site to the buccolingual alveolar ridge. Therefore, in this study, we have established a 5 mm keratinized mucosa covering the lingual side of alveolar crest with titanium mesh installation and measured the influence of keratinized mucosa by setting 5 mm keratinized mucosa in buccal side of alveolar crest for KM cases and 0 mm keratinized mucosa in buccal side of alveolar crest for LM cases. The physical properties of keratinized mucosa and non-keratinized mucosa was based on the measure of the thiel-embalmed cadavers provided by former research [22, 23, 35]. When the loading came from alveolar ridge, the forces was directly applied on keratinized mucosa in KM case or non-keratinized mucosa in LM case. The buccal keratinized mucosa reduced the displacement of soft tissue to form large contact with titanium mesh and decreased the maximum von Mises stress. Meanwhile, when the loading came from labial side of mucosa, the force was both applied on non-keratinized mucosa and delivered to keratinized mucosa in KM case or non-keratinized mucosa for LM case. The maximum von Mises stress was shifted to the junction of keratinized mucosa and lining mucosal. Although the value was slightly increased from 2.75 to 4.50 MPa due to the stiffness of keratinized tissue and less displacement, the von Mises stress value decreased from 2.75 to 2.50 MPa in non-keratinized mucosa and showed a more uniform stress distribution (Fig. 5). Taken together, those two situations both showed the existent of keratinized mucosa reduced stress concentration tendency, which might play an important role in preventing titanium mesh exposure.

The current study has several limitations, Firstly, the titanium mesh was set to be fixed, while the structure of titanium mesh would affect the distribution of stress in oral mucosa. However, to the best of knowledge, this is the first finite element model to analyze the displacement and stress distribution of oral soft tissue beyond porous titanium mesh and the results shows that the model has good simulation. Secondly, as computer simulation research, we found the keratinized mucosa in the buccal side of titanium mesh is critical for reducing stress in the soft tissue, and is effective against some indirect light force loading, which might lead to titanium mesh exposure. Future studies need further analyze the effect of keratinized mucosa in resisting to physiological load with large deformation models and validation from clinical.

## Conclusion

The outcomes demonstrate a successful finite element model was built to analyze the displacement and stress distribution between oral mucosa and titanium mesh. The mucosa was vulnerable under the increasing stress generated by the force from masticatory system, like chewing food. The adequate buccal keratinized mucosa width are critical factors in reducing the stress beyond the titanium mesh, which might reduce the titanium exposure rate.

## Data Availability

The datasets used and analysed during the current study available from the corresponding author on reasonable request.
